# Short- to mid-term results of minimally invasive lateral unicompartmental knee replacement: 133 cases in a non-designer series

**DOI:** 10.1007/s00402-023-04841-x

**Published:** 2023-03-14

**Authors:** Mustafa Hariri, Merlin Hagemann, Kevin-Arno Koch, Tobias Reiner, Benjamin Panzram, Christian Merle, Tobias Renkawitz, Tilman Walker

**Affiliations:** Department of Orthopaedics, Heidelberg University Hospital, Schlierbacher Landstrasse 200a, 69118 Heidelberg, Germany

**Keywords:** Lateral unicompartmental knee replacement, UKA, UKR, Fixed bearing, Partial knee arthroplasty

## Abstract

**Introduction:**

The aim of the current study was to demonstrate short- to mid-term survivorship as well as clinical outcome of lateral unicompartmental knee replacement (UKR) with a fixed-bearing (FB) design from a non-designer center using the Oxford Fixed Lateral prosthesis.

**Materials and methods:**

This single-center retrospective cohort study reports the results of 133 consecutive lateral FB-UKR. Survivorship analysis was performed with different endpoints and clinical outcome was measured using the Oxford-Knee-Score (OKS), American-Knee-Society-Score (AKSS-O), range-of-motion (ROM) and visual-analog-scale for pain (VAS).

**Results:**

There were two revision surgeries with conversion to total knee replacements (TKR) due to persistent pain resulting in a survival rate of 98.5% (95% CI 93.5–99.6) with a mean follow-up (FU) of 3.3 ± 1.8 years (range 1–8.5). All outcome scores, VAS and ROM showed a significant improvement at final FU (*p* < 0.001). The OKS improved from 26 ± 7.8 (range 11–45) preoperatively to 39 ± 8.3 (range 13–48), the AKSS-O from 49.2 ± 14.6 (range 18–90) to 81.8 ± 15.1 (range 40–100), the AKSS-F from 53 ± 23.7 (range 0–100) to 80.4 ± 21.4 (range 5–100) and the ROM from 118 ± 17 (range 90–160) to 134 ± 9.5 (range 100–155).

**Conclusions:**

The short- to mid-term results following lateral FB-UKR demonstrate a high survivorship and good clinical outcome from an independent series. We, therefore, suggest that FB-UKR is a safe treatment option for isolated lateral OA if sufficient surgical experience is provided.

**Level of evidence:**

Retrospective cohort study, level IV.

## Introduction

Isolated osteoarthrosis (OA) of the lateral compartment affects only 5–10% of patients with symptomatic knee OA [1]. Possible treatment options for advanced disease include distal femur osteotomy or knee arthroplasty [2, 3]. Unicompartmental knee replacement (UKR) can be performed in cases where the previously published indications and contraindications are met, otherwise total knee replacement (TKR) must be considered [4]. The advantages of UKR compared to TKR include a significantly lower rate of adverse events such as infections or blood loss [5]. In addition, patients following UKR tend to recover faster, have a shorter hospital stay, and achieve a better range-of-motion (ROM) compared to patients following TKR [6]. There are two different implant designs for UKR. The initial fixed-bearing (FB) design included a polyradial femoral component articulating on a flat all-polyethylene tibial component resulting in small contact areas and therefore in high contact pressure. Thus high failure rates due to polyethylene wear were reported [7]. Later, Goodfellow and O’Connor developed a mobile-bearing (MB) design to provide higher conformity between articular surfaces and to replicate tibiofemoral biomechanics in a more physiological manner than FB-UKR [8–10]. Therefore, many studies have reported excellent functional outcomes and high survivorship for MB-UKR used in the medial compartment [11–13]. In contrast, the initial results of MB-UKR in isolated lateral OA were disappointing with a survival rate of 82% at 5 years with bearing dislocation being the main cause for failure [14]. The reasons for the higher dislocation rate were thought to be the different anatomy and biomechanics of the lateral compartment compared to medial. While the medial collateral ligament (MCL) is tight in extension as well as in flexion and allows only about 2 mm of distraction, the lateral collateral ligament (LCL) is loose in flexion and allows up to 7 mm of distraction [15]. Additionally, the lateral femoral condyle accomplishes a higher backward movement in deep flexion than the medial condyle [16]. To address these specific differences, the implant was modified exclusively for the treatment of isolated lateral OA. This implant consists of a domed tibial plateau and a biconcave mobile bearing [17] to increase the entrapment of the bearing and consecutively reduce the risk of bearing dislocation [18, 19]. However, even with use of the new MB design, bearing dislocation still occurred with dislocation rates up to 8.5% after 5 years [20–22]. Therefore, the authors prefer the use of a FB design in isolated lateral OA. To date, there are only few contemporary studies reporting clinical results of lateral FB-UKR with small case numbers and a large variability in functional outcome and survivorship [1, 23–26].

The aim of the current study was to demonstrate the short- to mid-term results of the largest cohort of patients who underwent UKR using a FB design.

The hypothesis of this study was that lateral FB-UKR is a reliable treatment option with good short- to mid-term survivorship and high functional outcome in patients with isolated lateral OA.

## Material and methods

This current study is based on the retrospective analysis of prospectively collected data from a series of patients who underwent lateral UKR for isolated OA of the knee. In total, 143 UKR in 138 patients were performed between 2013 and 2020 using the Oxford Fixed Lateral prosthesis (Biomet Inc., Warsaw, Indiana, USA).

Inclusion criteria were a minimum age of 18 and signed informed consent; exclusion criteria were FU of less than 12 months.

Ethical approval was obtained by the institutional review boards of the University of Heidelberg (S-293-2021) and the study was conducted in accordance with the Helsinki Declaration of 1975, as revised in 2013. Informed consent was obtained from all participating patients.

The primary indication was severe OA of the lateral compartment with full thickness articular cartilage loss (“bone on bone”) or avascular necrosis of the femoral condyle. In all cases, clinical examination showed that the anterior cruciate ligament (ACL) as well as the MCL and LCL were functionally intact, the valgus deformity was manually correctable and there was no evidence of OA in the medial compartment on varus stress radiographs. OA of the patellofemoral joint was not considered a contraindication unless there was deep eburnation or bone grooving on the medial facet of the patella. The final assessment was performed intraoperatively to confirm the indication for UKR and if it was not appropriate, TKR was performed.

Rheumatoid arthritis, fixed valgus deformity, previous osteotomy, or a flexion deformity > 15° were considered contraindication.

In all cases, preoperative radiological assessment included radiographs as follows: AP and lateral weight bearing, varus/valgus stress, Rosenberg view and full-length standing AP.

All surgeries were performed using the minimally invasive surgical technique (MIS) through a lateral parapatellar approach without dislocation of the patella. Internal rotation of the tibial plateau and anatomical positioning of the femoral component were respected to avoid an elevation of the joint line. Bearing thickness was selected in full extension. In cases with a tight lateral compartment, we performed a resection of a 5–7 mm wide strip of bone from the lateral aspect of the patella to gain adequate exposure as described previously [27]. Depending on the bone quality the use of a cemented or uncemented fixation of the femoral component was chosen, whereas the tibial component was only available in a cemented version. An intravenous single-shot antibiotic (1.5 g cefuroxime) was administered perioperatively. All procedures were done by or under supervision of six well-trained surgeons with high experiences in knee replacement.

Postoperative rehabilitation is standardized for all patients. From the first postoperative day, immediate full weight bearing is possible. No restriction in active and passive knee movement was set. Discharge is followed by 3 weeks of inpatient or outpatient rehabilitation.

Survivorship analysis was performed with the endpoints “revision for any reason” and “any reoperation”. Revision surgery was defined as an operation in which at least one of the components was changed.

Clinical data were obtained through clinical examination by two of the authors (MH, MH) as part of a regular check-up to determine the functional and objective American Knee Society Score (AKSS-O and AKSS-F), the Oxford Knee Score (OKS) such as the range-of-motion (ROM). These regular check-ups are performed routinely for all patients receiving an arthroplasty at our institution 1, 3 and 5 years postoperatively. Standardized postoperative radiographs were aligned with fluoroscopic control to obtain views parallel to the tibial component in the AP-view and parallel to the femoral component in the lateral view. The radiographs were analyzed by two examiners (MH, MH) with focus on radiological signs of loosening of the components and progression of OA in the medial or retropatellar compartment.

Pain level was assessed using a visual analogue scale (VAS) ranging from 0 to 10 (0 = no pain, 10 = worst pain). Satisfaction postoperatively was evaluated by a numeric scale ranging from 1 (very satisfied) to 5 (unsatisfied).

Patients who were not able to attend the clinical FU were contacted by telephone for a structured interview to fill out the questionnaires to assess the OKS, level of pain as well as satisfaction with the prosthesis and possible complications. If necessary, additional information was gathered from general practitioners, orthopaedic specialists or relatives.

### Statistical methods

Data were recorded and analyzed using SPSS version 29.0 (SPSS Inc., Chicago, IL) and Graphpad Prism version 5.0 (Graphpad Software, San Diego, CA). Our primary endpoint was implant survival and secondary endpoints were clinical outcomes.

The empirical distribution of continuous variables was described using mean, standard deviation and range, possible differences between pre- and postoperative data were examined with the Wilcoxon Signed Rank test. Survivorship analysis was performed with the Kaplan–Meier estimator. The number at risk was provided for the mean FU timepoint and concludes the number of patients who have not yet experienced the event of interest or been censored. For all tests, the significance level was set at *p* < 0.05.

## Results

A total of 143 FB-UKR were implanted in 138 patients using the Oxford Fixed Lateral prosthesis between 2013 and 2020. Two patients (2 UKR) were lost-to-follow-up. Eight patients (8 UKR) died for unrelated reasons without any revision surgery during the study period. Information about death causes and about further surgeries were gathered through relatives.

The remaining 128 patients (133 UKR) were included in this study. Eighty-six patients (91 UKR) attended for a clinical FU and 42 patients (42 UKR) were contacted for a structured interview by telephone. The mean FU was 3.3 years ± 1.8 (range 1–8.5). In 131 cases, the underlying diagnosis was isolated OA of the lateral compartment from which three cases were posttraumatic and two patients (2 UKR) had a history of an avascular osteonecrosis of the femoral condyle. In 77 cases (57.9%) the femoral component was cementless while in 56 cases (42.1%) cemented femoral component was used. Patient demographics data are shown in Table 1.

**Table 1:** Clinical and demographic data of the study group

Demographics	
Patients (knees)	128 (133)
Mean follow-up in years ± SD (range)	3.3 ± 1.8 (1–8.5)
Mean age at time of surgery in years ± SD (range)	65 ± 12.3 (28–88)
Sex (%)	Female 97 (72.9%); male 36 (27.1%)
Operated side (%)	Left 41 (30.8%); right 92 (69.2%)
Mean body mass index (kg/m^2^) ± SD (range)	27.6 ± 5.3 (17.7–49.1)

*SD* standard deviation

### Survivorship analysis

**Fig. 1:**
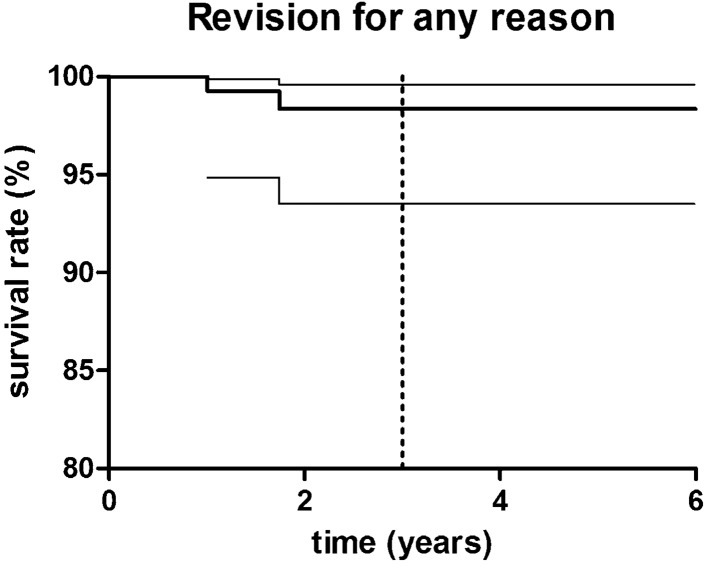
Kaplan–Meier survivorship curve with 95% confidence interval (CIs) (thin continuous lines) for “revision for any reason” as the endpoint. The 3-year survival was estimated at 98.5% (95% CI 93.5–99.6); dashed line presents mean follow-up timepoint of 3.3 years

There were two revision surgeries (1.5%) in the study group resulting in a survival rate with the endpoint “revision for any reason” of 98.5% (95% CI 93.5–99.6; number at risk: 71) at 3.3 years (Fig. 1). In both patients, the reason for revision surgery was a persistency of knee pain in the operated knee joint without any signs of progression of arthritis in the medial or retropatellar compartment, instability or low-grade infection. In both cases, cementless femoral component was used.

Revision surgery with conversion to TKR was performed at an external orthopaedic hospital in both cases.

**Fig. 2:**
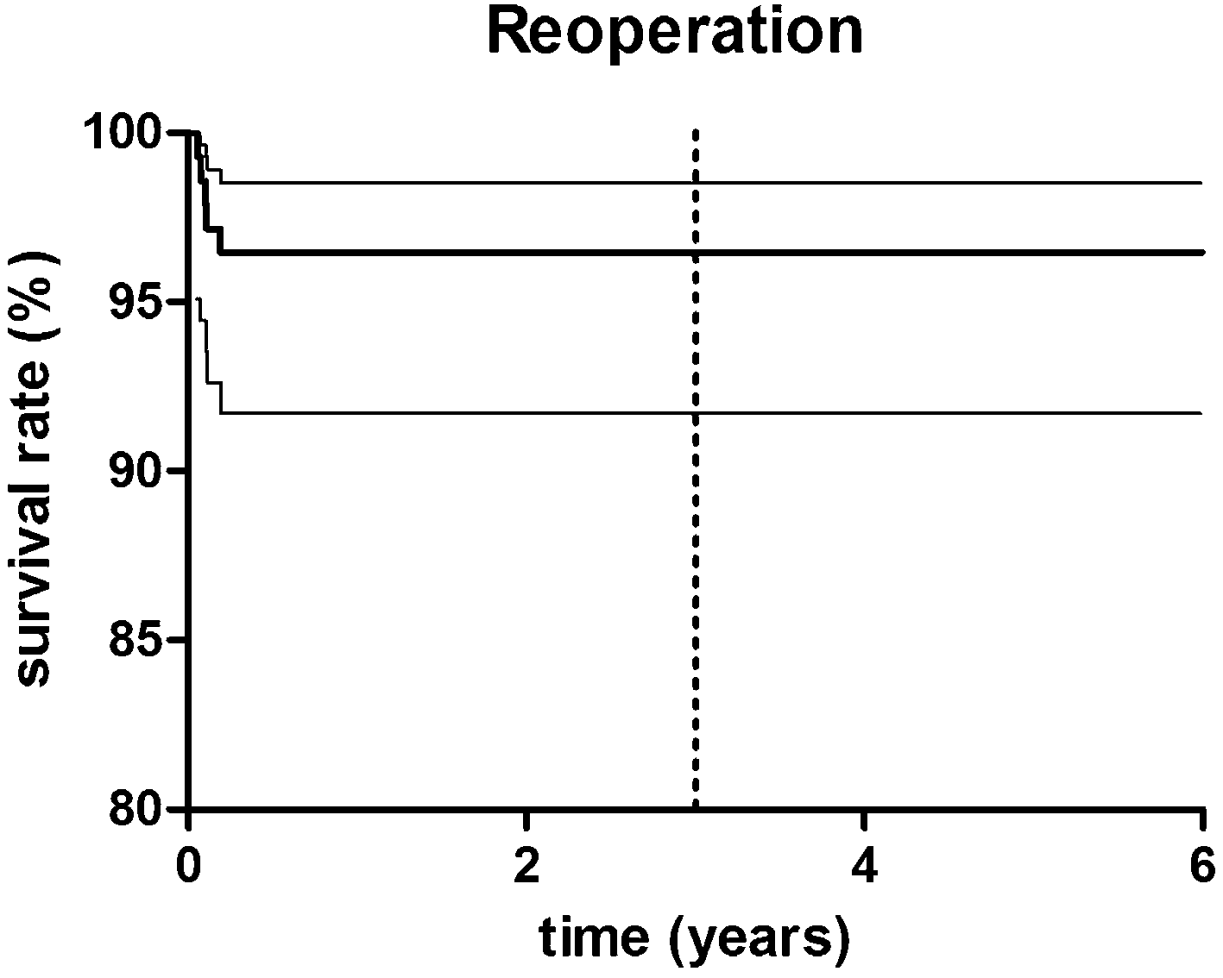
Kaplan–Meier survivorship curve with 95% confidence interval (CI) (thin continuous lines) for “reoperation” as the endpoint. The 3-year survival was estimated at 96.2% (95% CI 91.7–98.5); dashed line presents mean follow-up timepoint of 3.3 years

Reoperations were performed in 5 patients (5 UKR) (3.8%) due to several reasons resulting in a survival rate with the endpoint “reoperation” of 96.2% (95% CI 91.7–98.5; number at risk: 67) at 3.3 years (Fig. 2).

Two patients (1.5%) suffered from quadriceps-tendon rupture, from which one occurred during rehabilitation, and one was associated with a trauma three weeks postoperatively. Both patients received a tendon repair and showed excellent knee function at final FU with an OKS of 43 and 46, respectively. Another two patients (1.5%) suffered from aseptic wound healing disorder and received multiple lavages and debridements without any microbiological or histopathological signs of an infection. While at last FU one patient had an excellent outcome with an OKS of 44, the second patient only had a fair result with an OKS of 29 and an extension deficit of 10 degrees at final FU.

The last patient presented in the outpatient clinic five weeks postoperatively with an early-onset infection and evidence of E. coli in the microbiological samples. Following the DAIR (Debridement and implant retention)-procedure with multiple debridement and lavages, the implant could be retained. At final FU 12 months after surgery this patient had an excellent functional result with an OKS of 48.

### Clinical outcome

**Fig. 3:**
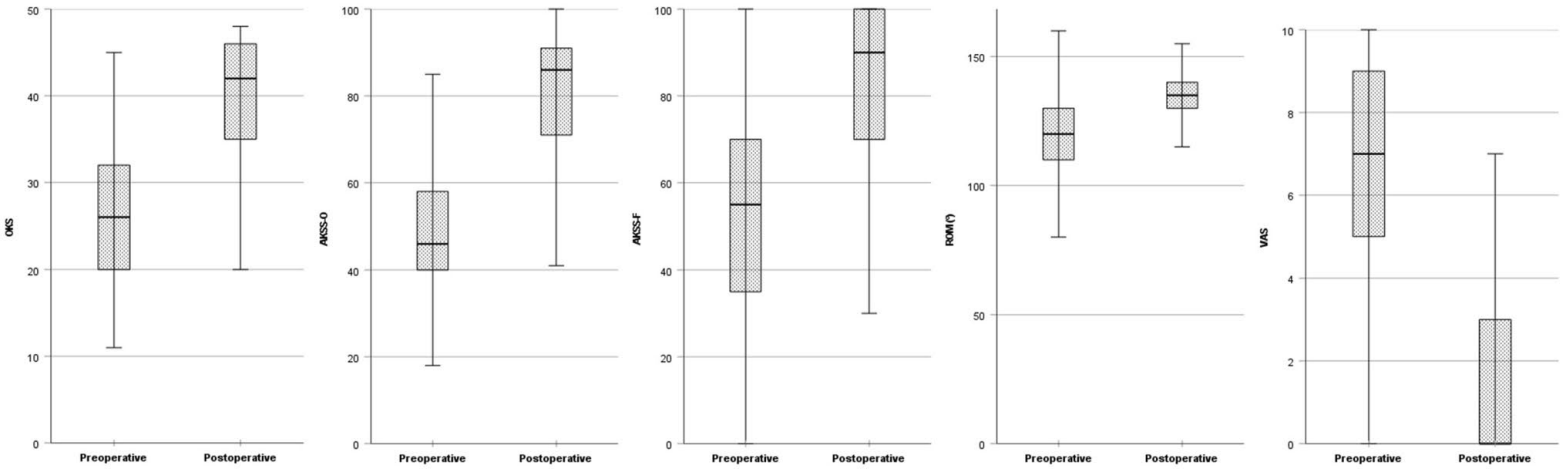
Clinical outcome scores, range-of-motion and visual-analogue-scale for pain preoperatively and at minimum 12 months follow-up. The differences were statistically significant (*p* < 0.001). OKS Oxford Knee Score, AKSS-O Objective American Knee Society Score, AKSS-F Functional American Knee Society Score, ROM range-of-motion, VAS Visual-analogue-scale for pain

All assessed postoperative parameters showed a significant improvement at final FU compared to preoperative as shown in Fig. 3. The mean ROM improved from 118° ± 17 (range 90–160) to 134° ± 9.5 (range 100–155) (*p* < 0.001).

The mean OKS showed an improvement from 26 ± 7.8 (range 11–45) to 39 ± 8.3 (range 13–48) (*p* > 0.001). According to the OKS-criteria, 52.9% had an excellent outcome (score > 41), 24.8% had a good outcome (34–41), 10.7% had a fair outcome (27–33) and 11.6% had a poor outcome (< 27) at final FU.

The mean AKSS-O improved statistically significant from 49.2 ± 14.6 (range 40–100) to 81.8 ± 15.1 (range 5–100) (*p* > 0.001). According to the AKSS-O criteria, 61.6% had an excellent outcome (85–100), 18.8% had a good outcome (70–84), 9.8% had a fair outcome (60–69) and 9.8% had a poor outcome (< 60).

The mean pain level on VAS improved from 7 ± 2.2 (range 0–10) to 1.6 ± 2.3 (range 0–10).

Altogether 91.3% of the patients were satisfied or highly satisfied with the outcome, 5.5% were fairly satisfied and 3.1% were unsatisfied. Clinical results are shown in Table 2.

**Table 2:** Clinical results

	Preoperative mean ± SD (range)	Postoperative mean ± SD (range)	
OKS***	26 ± 7.8 (11–45)	39 ± 8.3 (13–48)	
AKSS-O***	49.2 ± 14.6 (18–90)	81.8 ± 15.1 (40–100)	
AKSS-F***	53 ± 23.7 (0–100)	80.4 ± 21.4 (5–100)	
ROM***	118 ± 17 (90–160)	134 ± 9.5 (100–155)	
VAS***	7 ± 2.2 (0–10)	1.6 ± 2.3 (0–10)	

OKS Oxford Knee Score, AKSS-O Objective American Knee Society Score, AKSS-F Functional American Knee Society Score, ROM Range-of-motion, VAS Visual Analogue Scale, SD standard deviation

***p < 0.001

### Radiological outcome

Radiographic analysis was performed for all patients who attended to the outpatient clinic for FU (91 UKR). In one female patient, OA progression in the medial compartment was diagnosed 2.7 years after UKR implantation. This patient reported about persistent knee pain and was unsatisfied with the result. Conversion to TKR was recommended, but the patient rejected revision at last FU. In all other cases, no signs of loosening or OA progression was observed.

## Discussion

The main findings of the current study are high implant survivorship of 98.5% with the endpoint “revision for any reason” for lateral UKR and satisfactory clinical outcomes in about 80% of the patients in short- to mid-term in a non-inventor series.

Due to the low incidence of isolated lateral OA, there are only few publications that have investigated the clinical results and survivorship of lateral FB-UKR. Moreover, different implants were analyzed allowing only limited comparison between them. Nevertheless, the survival rate of the current study group is comparable to these previously reported results from non-designer series with survival rates ranging from 91.4 to 100% in long-term FU [1, 23, 25, 26].

Recently, Asadollahi et al. reported first results from a designer series of 130 implants with the Oxford Fixed Lateral prosthesis. Survival rate at 4 years for implant failure was 100% [28].

While the current study demonstrated a high survivorship, a recent report from the Dutch Arthroplasty Register showed a revision rate of 8.3% after 5 years for 168 lateral FB-UKR [29]. Critics of UKR often refer to the discrepancy between outcomes of independent series, inventor studies and registry studies. A main reason for this is thought to be the disparity in surgical experience in UKR [5]. Registry studies include results from centers with low surgical volume and surgeons performing fewer than the recommended number of 10–12 UKR per year, which is associated with higher revision rates [30, 31].

One patient of the study population suffered from symptomatic OA progression without revision to TKR due to personal preference. The cause of OA progression after lateral UKR is partly attributed to overcorrection of valgus [32, 33]. Since the indication for revision to TKR was already present, full-length standing AP radiograph was not performed postoperatively and malalignment was, therefore, not analyzed. OA progression, mostly in late years, seems to be the most common reason for revision after FB-UKR as reported by several systematic analysis [29, 33, 34].

Bearing design seems to influence survivorship of lateral UKR. Burger et al. demonstrated favorably survivorship of lateral FB-UKR compared to MB-UKR [35]. In contrast, Tay et al. reported about similar revision rates between both designs when metal-backed and all-polyethylene FB designs were analyzed together, but after subgroup analysis metal-backed FB designs showed lower revision rates compared to other designs [33]. A recent study by Fratini et al. confirmed, that metal-backed FB-UKR has lowest revision rate for lateral UKR [36].

As described previously, two patients suffered from a quadriceps tendon rupture postoperatively [27]. After modification of the surgical technique [27], we haven’t observed this complication anymore. In addition, we did not notice any complication due to the bone resection. Therefore, we believe this procedure is effective in preventing extensive soft tissue damage and should be performed in patients with limited exposure of the lateral knee joint.

In about 80% of all cases in the present study, the functional outcome is good to excellent. The mean AKSS-O of 81.8 and mean OKS of 39 postoperatively are comparable to previously reported results for lateral FB-UKR [26, 28] as well as for MB-UKR with the Oxford domed lateral prosthesis [17, 22, 37, 38]. However, about 20% of our patients had only fair to poor outcome. The reasons for these results are multifactorial. In some patients, comorbidities impaired response to the AKSS and OKS, similar to what was reported in a study by Newman et al. about lateral MB-UKR [19]. Other patients complained about persistent knee pain. After ruling out objective causes such as aseptic loosening, infection, instability, or OA progression in these cases, conservative treatment was preferred. Many cohort studies use only mean values to describe clinical outcomes in UKR. The proportion of fair results is often not reported, limiting comparison of our results with other studies. In a recent study by Asadollahi et al. 11% of patients had fair or poor outcomes following FB-UKR, which they attributed to persistent knee pain or other medical conditions [28].

In general, the management of dissatisfied patients without objective causes remains difficult as the threshold for revision surgery is much lower for UKR than for TKR. Murray et al. reported, that revision surgery was six times more likely in patients with similar OKS scores of less than 20 after UKR than after TKR [39]. In addition, outcomes after revision surgery for unexplained pain appear to be worse than after revision for an identified cause [40].

To our knowledge, this study is the largest series reporting survival rates and clinical results of the Oxford Fixed Lateral prosthesis. Our findings demonstrate good to excellent outcome parameters in about 80% of the patients in short- to mid-term following lateral FB-UKR at an independent center and therefore suggest that the results from the designer center are transferable [28].

This study has several limitations, such as limited sample size and the retrospective study design. Additionally, patient-related outcome parameters were collected at different timepoints ranging from 1 to 8.5 years. Data collection at the same timepoint for all patients would have strengthen the presented data. Furthermore, selection bias is present as approximately one third of the patients did not attend to our outpatient clinic and were only assessed by telephone. Moreover, radiographic analysis was missing in these patients, which is another limitation of this study.

## Conclusion

The short- to mid-term results following lateral FB-UKR with the Oxford Fixed Lateral prosthesis show a high survivorship and good clinical outcome in an independent series. We, therefore, suggest that FB-UKR is a safe treatment option for isolated lateral OA when sufficient surgical experience is provided.

## Data Availability

The datasets used and analyzed during the current study are available from the corresponding author on reasonable request.

## Abbreviations

ACL: Anterior cruciate ligament
AKSS-F: Functional American Knee Society Score
AKSS-O: Objective American Knee Society Score
FB: Fixed-bearing
FU: Follow-up
LCL: Lateral collateral ligament
MB: Mobile-bearing
MCL: Medial collateral ligament
MIS: Minimally invasiv surgical technique
OA: Osteoarthrosis
OKS: Oxford knee score
ROM: Range-of-motion
TKR: Total knee replacement
UKR: Unicompartmental knee replacement
VAS: Visual-analog-scale for pain
